# Influence of Particle Size and Hot-Pressing Parameters on Mechanical Properties of Bamboo-Based Composite Materials

**DOI:** 10.3390/biomimetics10030156

**Published:** 2025-03-03

**Authors:** Jun Lu, Kuichuan Sheng, Jie Chen, Xumin Ding, Zichao Wen, Sha Li

**Affiliations:** 1 Mechanical College, Shanghai Dianji University, Shanghai 201306, China; lujun@sdju.edu.cn (J.L.);; 2 Department of Agricultural Engineering, Zhejiang University, Hangzhou 310058, China; 3 Higher Vocational and Technical College, Shanghai Dianji University, Shanghai 200240, China

**Keywords:** biocomposites, hot press moulding, particle size, mechanical properties

## Abstract

A novel series of biocomposites was prepared through the utilisation of hot-pressing and blending methods, utilising bamboo particles of varying sizes and a zein solution. The influence of particle size and the parameters of hot pressing on the mechanical properties of the composites was investigated through the application of an alkali solution to pre-treat the bamboo particles and the employment of ultra-high pressure to pre-handle the zein solution. Four response surface models were established to optimise the processing parameters based on mechanical testing experiments and quadratic regression analysis. The influencing factors were moisture content, press temperature, and press time, and the responses were modulus of rupture (MOR), modulus of elasticity (MOE), tensile strength (TS) and 2 h thickness swelling rate (2h-TSR). The findings indicated that the TS of composites reached a maximum value of 17.5 MPa with a bamboo particle size of 40 mesh; the MOR and MOE of composites reached a maximum value of 28.72 MPa and 2669.75 MPa when the bamboo particle size was 60 mesh; regarding the 2h-TSR of composites the lowest value of 5.8% was obtained for 80-mesh bamboo particle size. The optimum moulding process parameters were obtained with moisture content ranging from 14% to 16%, press temperature ranging from 170 °C to 175 °C, and press time ranging from 12 min to 15 min, respectively.

## 1. Introduction

Bamboo, which is found in abundance in China and many other Asian countries, has the potential to be exploited for the design and development of polymer composites. The structural variation, mechanical properties, extraction of fibres, chemical modification, and thermal properties of bamboo fibres have rendered it versatile for use in the composite industry [1].

Zein has been used as fibres, films, and adhesives. Its potential commercial value is attributable to its edibility and biodegradability, which underpin its applications in food, medicine, and the chemical industry. Currently, zein is being studied as a potential bio-based polymeric material [2]. As two kinds of green materials, bamboo and zein have attracted greater attention and been explored further [3,4,5].

In the context of hot-pressing and blending preparation of biocomposites, the size of the powder raw particles, size distribution and bulk density of compacted sampless have been demonstrated to exert a significant influence on the panel heating performance [6,7,8,9,10]. Furthermore, the process of bamboo particle hot pressing has been shown to be a complex polymer thermoplastic melting and densification procedure, which is associated with a multitude of factors. A reasonable match of temperature, pressure and time is identified as a key factor [11,12,13,14,15]. This is also one of the key problems in exploring the toughness design of bamboo fibre-reinforced resin matrix composites. As for the influence of particle size and hot-pressing parameters on the panel heating properties of biocomposites, many foreign scholars have performed related research. For instance, Q Jiang, N Reddy and Y Yang have reported a novel method of cross-linking electrospun zein fibres using citric acid as a non-toxic cross-linker to enhance the water stability and cytocompatibility of zein fibres for tissue engineering and other medical applications (Jiang et al., 2010) [16]. The investigation further encompasses the incorporation of zein, a water-insoluble maize protein, in the coating process. This approach was employed to achieve the objective of producing low-toxicity selenite-loaded selenite supplements, with the overarching goal being to enhance the antioxidant properties of the final product. This study was conducted by Luo, Y., Zhang, B., Cheng, W. H., and Wang, Q. (Luo et al., 2010) [17,18,19]. The study of wooden powder raw materials as compressible continuous units has given rise to a series of theories and rules, including the Shima model of porous materials and Kim’s rules, which are based on thermo-elastic plasticity theory [20,21,22,23]. In China, Wu (2010) [24,25] proposed a novel concept regarding the warm compaction forming process of wood powder. It was found that reed stalk powder, poplar powder and bamboo powder exhibited superior formability at room temperature with increasing particle size, while pine powder and cotton stalk powder demonstrated the opposite behaviour. These findings highlight the distinct advantages and potential of reed stalk powder and poplar powder in terms of comprehensive properties, including size stability, moldability, stretching resistance, and impact resistance [26]. It is acknowledged that novel biocomposites of bamboo particles and corn protein can be prepared by hot-pressing and mixing methods. However, further research is required to determine the impact of the particle size of bamboo particles and the hot-pressing parameters on the mechanical properties of these composites.

Research at the intersection of bamboo-based composites and biomimicry focuses on optimising material properties by mimicking natural biological structures. In recent years, scientific research has found that the unique hierarchical vascular bundle structure (gradient density distribution, hollow fibre arrangement) of bamboo provides a biomimetic template for composite design. For example, by simulating the mechanical transfer mechanism of bamboo joint walls, researchers have developed a new composite material with an impact-resistant layered structure whose flexural strength can be increased by more than 30% (Chen, et al., 2022) [27]. In terms of interfacial biomimicry, inspired by the synergistic effect of bamboo fibres and thin-walled cells, the research team used 3D printing to construct a biomimetic honeycomb core layer structure, which, combined with surface micro-nano-topological modification, improved the interfacial bond strength of the composites by 45% (Zhang, Y., et al., 2021) [28]. Another study mimicked the surface microgroove structure of bamboo leaves to develop bamboo-plastic composites with a self-cleaning function and a contact angle of 152°, which significantly improves outdoor durability (Li, J., et al., 2023) [29].

The development trend in this field shows two directions: firstly, the development of smart responsive materials such as humidity-driven deformation composites through multi-scale biomimetic design (molecular-microscopic-macroscopic) and secondly, the optimisation of the sustainable preparation process by combining with life cycle analysis, which has already achieved a biodegradation rate of more than 80% of bamboo-based composites (Wang, H., et al., 2022) [30]. These breakthroughs provide new eco-friendly material solutions for green buildings, flexible electronic devices and other fields.

In this paper, novel biocomposites with bamboo particles of varying size and zein solution were prepared by applying hot-pressing technology and the blending method. In order to investigate the influence of particle size and process parameters on the performance of panel compacting, different sizes of bamboo particles, moisture contents of the panel, press temperatures, and press times were analysed to establish a response surface model for optimum processing conditions. The findings of this study offer a theoretical foundation and practical guidance for the hot-pressing and moulding process performance of bamboo/zein composites.

## 2. Materials and Methods

### 2.1. Materials and Instruments

#### 2.1.1. Experimental Material

The following substances were used in the production of the substance under investigation: bamboo particles (bamboo raw materials, provided by Lin’an Bamboo Textiles Co., Ltd., Lin’an, China); zein (the protein content is approximately 91%, provided by Rixing Pharmaceutical Necessities Co., Ltd., Gaoyou, China); sodium hydroxide (Xilong Chemical Co., Ltd., Shantou, China); deionized water (pure water supply station, provided by College of Environment and Resources, Zhejiang University, Hangzhou, China); ethanol (analytical reagent, provided by Si-nopharm Group Chemical Reagent Co., Ltd., Shijiazhuang, China); and mould release agents (provided by Shenzhen Saiya Aerosol Co., Ltd., Shenzhen, China).

#### 2.1.2. Main Instruments

The following pieces of equipment were utilised in the experiment: ultra-high-pressure equipment (UHTF-750 MPA, Baotou Kefa High Pressure Science and Technology Co., Ltd., Baotou, China); a computer controlled electronic universal testing machine (CNT-4204, American MTS Co., Ltd., Minneapolis, MN, USA); a hot-press moulding machine (GT-7014-A50C, GOTHCH, Dongguan Co., Ltd., Dongguan, China); a mixer (B-20, Panyu, Guangzhou Lifeng Co., Ltd., Guangzhou, China); a precision force electric mixer (JJ-1, Jiangnan Co., Ltd., Jintan, China); a flow-through Chinese medicine grinder (YF3-1, Yongli Pharmaceutical Machinery Co., Ltd., Ruian, China); an electric oven (DHG-9077A, Jinghong Experimental Equipment Co., Ltd., Shanghai, Company); electronic digital callipers (Guanglu Measuring Instrument Co., Ltd., Guilin, China); an analysis balance (JA-5003N, Shuangjie Test Instrument Co., Ltd., Changshu, China); and a forming mould (made by Bi-oenergy & Biomaterials laboratory, College of Biosystems Engineering and Food Science, Zhejiang University, Hangzhou, China). The mould’s internal dimensions were 152 × 152 × h mm^3^, where h denotes the thickness of the composites.

### 2.2. Experimental Method

#### 2.2.1. Selecting Particles Size of Bamboo Powder and Its Alkali Treatment

The selection of bamboo powder particles was conducted by grinding bamboo sawdust into powder, with the particles sized at 20 mesh (approximately 830 μm), 40 mesh (approximately 380 μm), 60 mesh (approximately 250 μm), and 80 mesh (approximately 180 μm), respectively. The selection of the bamboo powder particle size involved the grinding of bamboo sawdust into powder, followed by the filtration of the particles through sieves with mesh numbers of 20, 40, 60, 80 and 100, respectively. This resulted in the production of five distinct bamboo particle sizes, which were subsequently subjected to the drying process in an oven.

The experiment involved the treatment of bamboo particles (60-mesh) with varying concentrations of NaOH solutions (1%, 2%, 3%, 5%, 10%) for a duration of 15 min at a temperature of 20 °C. The samples were then subjected to agitation to ensure complete mixing. The mixture was formed by stirring and mixing 60-mesh bamboo particles soaked in NaOH solution at 1 per cent with 60-mesh bamboo particles soaked in NaOH solution at 2 per cent, 3 per cent, 5 per cent and 10 per cent. Then, the mixture was put into a crucible for drying until the moisture content reached 3% in the drying oven at 75 °C. The dried mixture was transferred to another crucible for further processing.

#### 2.2.2. Preparation of Zein Solution and Its Ultra-High-Pressure Treatment

The preparation of the zein solution commenced with the amalgamation of anhydrous ethanol and deionised water in a volume-fractional ratio of 85:15 to yield an ethanol solution. This solution was subsequently transferred into a 100 mL beaker, whereupon 30 g of zein powder was incorporated and the mixture was subjected to stirring for a duration of 1.5 h. This process was undertaken until complete dissolution of the zein powder within the solution occurred. The resulting solution possessed a final concentration of 300 g/L of zein.

Ultra-high-pressure treatment of zein solution: The zein solution was prepared and divided into six groups. Following sealing in polyethylene plastic bags, the solutions were placed in a high-pressure container. Pressures of 100, 200, 300, 400, 500 and 600 MPa were applied to the six groups of solution at 25 °C, respectively. The duration of pressure treatment for each group was 10 min, corresponding to the pressure levels specified. The experimental data, expressed as the mean of triplicate measurements, was analysed and averaged unless otherwise indicated.

#### 2.2.3. Preparation of Bamboo/Zein Composites

The procedure involved the amalgamation of bamboo particles (following an alkali treatment) with a zein solution (subsequent to a high-pressure treatment). The precise mass of bamboo particles was determined, and they were transferred into a mixer. The zein solution, which had undergone a high-pressure treatment, was then added to the mixer in an even manner. The mixture was subjected to continuous agitation for a minimum of 15 min, with the objective of ensuring a uniform distribution of bamboo and zein particles.

The adjustment of the moisture content was achieved by placing the bamboo particles into a tray and subsequently measuring their wet weights. Between 103 and 2 °C, 2–4 g of bamboo particles was collected in a drying oven for moisture content testing. The total mass of bamboo particles was calculated, and then the dry quality of bamboo particles was obtained by means of low-temperature drying. It is important to note that the desired moisture content can be adjusted by controlling the mass of bamboo particles after drying.

In the hot-pressing moulding process, the bamboo particles were amalgamated with the zein solution within a bespoke forming mould. A 1 mm thick plate was positioned on the panel’s surface, with aluminium foil placed between the panel and the plate. An adequate quantity of mould release agent was then dispensed onto the aluminium foil to prevent adhesion between the panel and the bamboo particles. A moderate amount of bamboo particles was installed in the panel mould to regulate the density of the composites prior to their introduction into the hot-press machine. The hot-pressing moulding conditions were established through thermoforming as follows: a press temperature of 170 °C, a press pressure of 6 MPa, and a press time of 8 min. Each test was replicated thrice, and the composites were placed in the incubator for a duration of 48 h.

#### 2.2.4. Mechanical Property and Water Resistance Tests

In the mechanical property test, the composite sample was sawn into strips measuring 15.24 cm by 2.5 cm. These were then measured using a computer-controlled electronic universal testing machine.

Water resistance: The sample was cut into strips measuring 2.5 cm × 2.5 cm, and its thickness was measured. The strips were then immersed in a sink with a pH value of 7 ± 1 and a temperature of 20 ± 2 °C. After soaking for 2 h, the water on the surface of the sample was wiped off, and the thickness of the sample strip was measured within 10 min.

#### 2.2.5. Establishing Response Surface Model of Mechanical Properties of Composites

The indicator of mechanical properties was regarded as response value, which included the modulus of rupture (MOR), modulus of elasticity (MOE), tensile strength (TS) and 2 h thickness swelling rate (2h-TSR), and the parameters of hot pressing were identified as a factor that influenced the mechanical properties of the materials. The influence of moisture content, press temperature and press time was taken into consideration. A mathematical regression model was established to predict the mechanical properties of zein/bamboo composites. The model was then used to generate surface plots using test design software (SAS v9.0). The indicators and parameters of the response surface model are shown in Table 1.

## 3. Results and Discussion

### 3.1. Appearance Changes of Bamboo Powder Treated with Alkali

**Figure 1:**
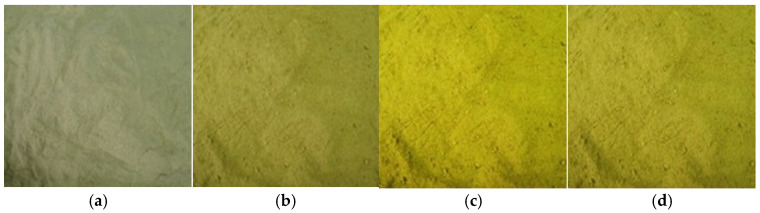
Appearance of bamboo particles as an effect of concentration of NaOH (**a**) untreated; (**b**) 2%NaOH-treated; (**c**) 5%NaOH-treated; (**d**) 10%NaOH-treated.

Following treatment with an alkali (NaOH), a marked change in the colour of bamboo particles was evident, as illustrated in Figure 1. The colour of bamboo powder treated with alkali exhibited yellow hues in varying degrees. These colour alterations can be attributed to the precipitation of hemicellulose.

### 3.2. Effect of Particle Size of Bamboo Powder on the Mechanical Properties of Composites

#### 3.2.1. Effect of Particle Size of Bamboo Powder on the Modulus of Rupture (MOR) of Composites

**Figure 2:**
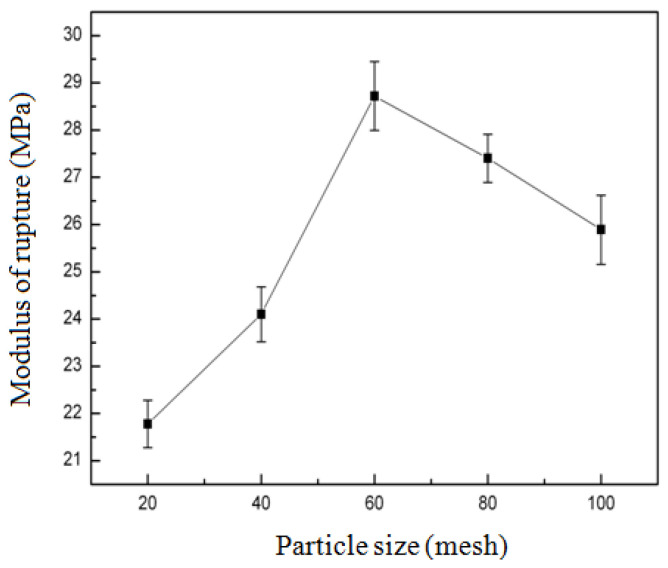
Effect of particle size of bamboo powder on the modulus of rupture (MOR) of composites.

As demonstrated in Figure 2, the effect of bamboo powder particle size on the modulus of rupture (MOR) of composites was investigated. The particle size of bamboo powder ranged from 20 mesh to 100 mesh, and the MOR of composites exhibited an initial increase followed by a subsequent decrease. Notably, when the particle size of bamboo powder was 20 mesh, the MOR of composites reached 21.78 MPa. Conversely, at a particle size of 60 mesh, the MOR of composites attained its maximum of 28.72 MPa. As the particle size of bamboo particles increased from 20 mesh to 60 mesh, it was evident that the higher the mesh number, the more pore holes there are and the stronger the bonding with zein, and there is a consequent increase in MOR. However, after increasing the particle size from 60 mesh to 100 mesh, the particles were finer, and the prepared material became loose, with consequent decrease in MOR.

#### 3.2.2. Effect of Particle Size of Bamboo Powder on the Modulus of Elasticity (MOE) of Composites

**Figure 3:**
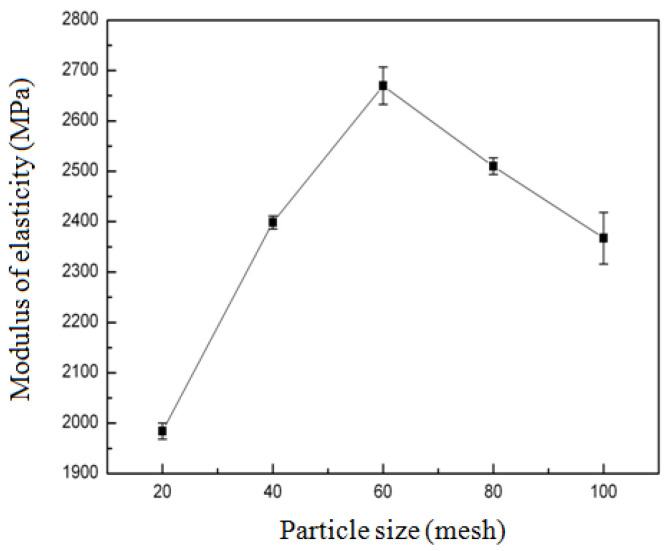
Effect of particle size of bamboo powder on the modulus of elasticity (MOE) of composites.

As demonstrated in Figure 3, the effect of bamboo powder particle size on the modulus of elasticity (MOE) of composites was investigated. The particle size of bamboo powder ranged from 20 mesh to 100 mesh, and the MOE of composites exhibited an initial increase, followed by a subsequent decrease. At a particle size of 20 mesh, the MOE of composites was recorded at 1984.3 MPa. However, when the particle size of bamboo powder was increased to 60 mesh, the MOE of the composites reached its maximum of 2669.75 MPa. The particle size of bamboo particles increased from 20 mesh to 60 mesh; the higher the mesh number, the more pore holes and the stronger the bonding with zein, with consequent increase in MOE; however, after increasing the particle size from 60 mesh to 100 mesh, the particles were finer, and the prepared material became loose, with a consequent decrease in MOE.

#### 3.2.3. Effect of Particle Size of Bamboo Powder on the Tensile Strength (TS) of Composites

**Figure 4:**
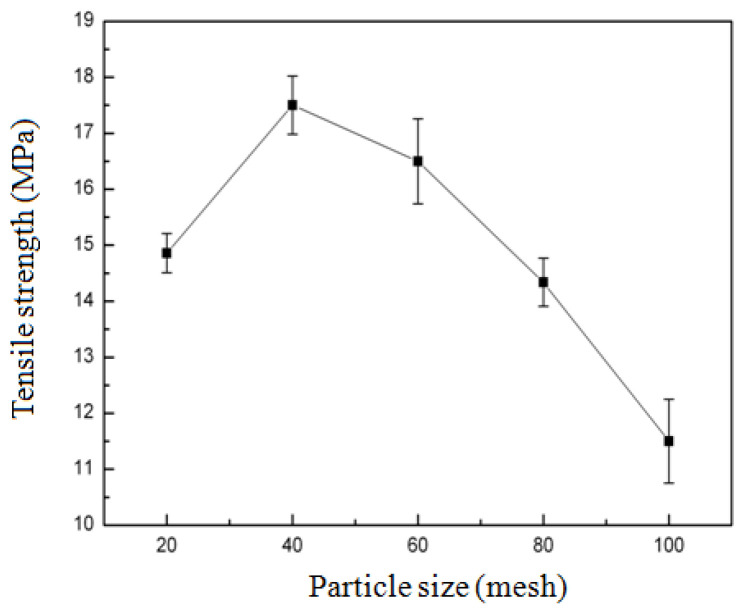
Effect of particle size of bamboo powder on the tensile strength (TS) of composites.

As demonstrated in Figure 4, the effect of bamboo powder particle size on the tensile strength (TS) of composites was investigated. The particle size of bamboo powder ranged from 20 mesh to 100 mesh, with an initial increase in TS followed by a subsequent decrease. Notably, when the particle size of bamboo powder was set at 100 mesh, the TS of the composites was recorded at 11.5 MPa. Conversely, at a particle size of 40 mesh, the TS of composites attained its maximum of 17.5 MPa. The particle size of bamboo powder ranges from 20 mesh to 40 mesh, with smaller particle sizes resulting in tighter combinations with zein and increased tensile strength (TS). However, from 20 mesh to 100 mesh, the finer the particles, the more the composite material becomes loose, and the TS decreases.

#### 3.2.4. Effect of Particle Size of Bamboo Powder on the 2 h Thickness Swelling Rate (2h-TSR) of Composites

**Figure 5:**
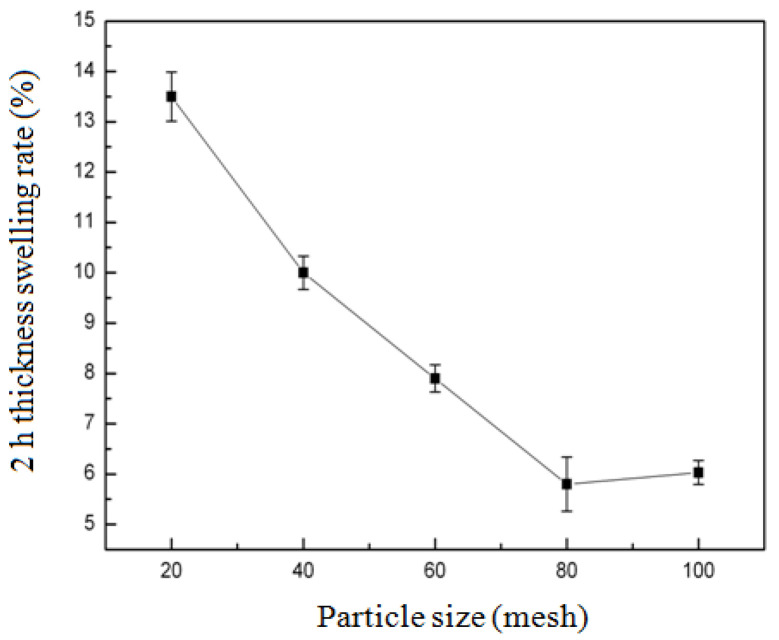
Effect of particle size of bamboo powder on the 2 h thickness swelling rate (2h-TSR) of composites.

As demonstrated in Figure 5, the effect of bamboo powder particle size on the 2 h thickness swelling rate (2h-TSR) of composites was investigated. The particle size of bamboo powder ranged from 20 mesh to 100 mesh, and the 2h-TSR of composites exhibited a decrease initially, followed by an increase. Notably, when the particle size of bamboo powder was 20 mesh, the 2h-TSR of composites was recorded at 13.5%. When the particle size of bamboo powder was 80 mesh, 2h-TSR of composites reached its minimum of only 5.8%. The particle size of bamboo powder was increased from 20 mesh to 80 mesh, as the higher the mesh number, the smaller the particle size. It was hypothesised that the more porous the prepared composite material, the lower its moisture content, and that the 2 h thickness swelling ratio (2h-TSR) would decrease with the increase in mesh number. However, when the mesh number increases from 80 to 100, the material becomes loose and the 2h-TSR increases slightly.

### 3.3. Influence of Hot-Pressing Parameters on Mechanical Properties of Composites

#### 3.3.1. Influence of Moisture Content and Hot-Pressing Parameters on the Modulus of Rupture (MOR) of Composites

In order to design the experiments, response surface analysis was employed, and the results were simulated by means of statistical software (SAS v9.0) to establish a regression equation (Model 1). The following formula (Formula (1)) can be used to obtain the polynomial quadratic equation of modulus of rupture (MOR):

(1) $$\begin{array}{cc} Y_{1}= & -1681.09+9.01X_{1}+18.01X_{2}+11.31X_{3}-0.20{X_{1}}^{2}-0.02X_{1}X_{2}\\ & +0.02X_{1}X_{3}-0.05{X_{2}}^{2}-0.05X_{2}X_{3}-0.14X_{3}^{2} \end{array}$$

**Figure 6:**
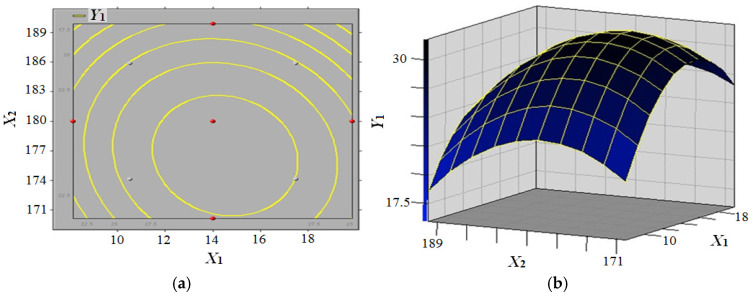
Response surface model of panel moisture content and press temperature effect on the modulus of rupture (MOR) of composites (**a**) MOR contour curve; (**b**) MOR contour surface.

The response surface model illustrating the effect of panel moisture content and press temperature on the modulus of rupture (MOR) of composites is presented in Figure 6. The MOR (*Y*_1_) exhibited an initial increase, followed by a subsequent decrease, with an increase in panel moisture content (*X*_1_), under constant press temperature (*X*_2_) and press time (*X*_3_) conditions of 10 min. Conversely, MOR (*Y*_1_) demonstrated an initial increase, followed by a subsequent decrease, with an increase in press temperature (*X*_2_), when panel moisture content (*X*_1_) was constant within the experimental level. The numerical solution of the equation (Formula (1)) yielded the contour curves (Figure 6a) and contour surface (Figure 6b). The response surface method was employed to analyse the simulation results, thereby identifying the optimisation parameters. The results obtained demonstrated that the optimal conditions for achieving the maximum MOR (*Y*_1_) of 31.86 MPa were as follows: a panel moisture content (*X*_1_) of 14%, a press temperature (*X*_2_) of 175 °C, and a press time (*X*_3_) of 12 min.

#### 3.3.2. Influence of Moisture Content and Hot-Pressing Parameters on the Modulus of Elasticity (MOE) of Composites

The polynomial quadratic equation (Model 2) was solved by employing software SAS v9.0 in conjunction with the response surface methodology. The polynomial quadratic equation of modulus of elasticity (MOE) can be obtained as follows (Formula (2)):

(2) $$\begin{array}{cc} Y_{2}= & -160891.00+819.04X_{1}+1741.07X_{2}+793.07X_{3}-17.66{X_{1}}^{2}-1.92X_{1}X_{2}\\ & +3.07X_{1}X_{3}-4.75{X_{2}}^{2}-3.25X_{2}X_{3}-11.45{X_{3}}^{2} \end{array}$$

**Figure 7:**
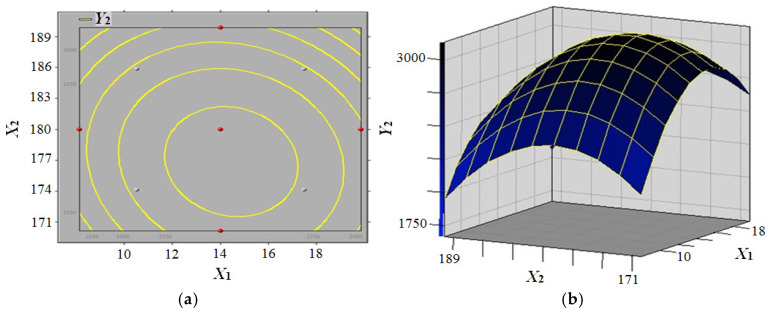
Response surface model of panel moisture content and press temperature effect on the modulus of elasticity (MOE) of composites (**a**) MOE contour curve; (**b**) MOE contour surface.

The response surface model of panel moisture content and press temperature’s effects on the modulus of elasticity (MOE) of composites is illustrated in Figure 7. The figure demonstrates that MOE (*Y*_2_) initially increases and subsequently decreases with increasing panel moisture content (*X*_1_) when press temperature (*X*_2_) and press time (*X*_3_) are constant for 10 min. Conversely, MOE (*Y*_2_) increases initially and then decreases with increasing press temperature (*X*_2_) when panel moisture content (*X*_1_) is constant within the experimental level. The parameters of the hot-pressing process were optimised by single-factor experiments and response surface analysis, and the results showed that the optimal conditions were panel moisture content (*X*_1_) of 14%, press temperature (*X*_2_) of 175 °C and press time (*X*_3_) of 12 min. When these conditions are met, MOE (*Y*_2_) can reach its theoretical maximum of 3139.36 MPa.

#### 3.3.3. Influence of Moisture Content and Hot-Pressing Parameters on the Tensile Strength (TS) of Composites

The polynomial quadratic equation (Model 3) was solved by employing software SAS v9.0 in conjunction with the response surface methodology. The polynomial quadratic equation of tensile strength (TS) can be obtained as follows (Formula (3)):

(3) $$\begin{array}{cc} Y_{3}= & -581.08+7.52X_{1}+5.93X_{2}+4.81X_{3}-0.11{X_{1}}^{2}-0.03X_{1}X_{2}\\ & +0.001X_{1}X_{3}-0.02{X_{2}}^{2}-0.02X_{2}X_{3}-0.03{X_{3}}^{2} \end{array}$$

**Figure 8:**
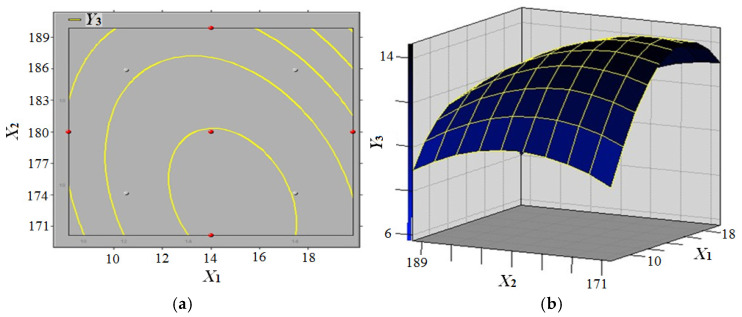
Response surface model of panel moisture content and press temperature effect on the tensile strength (TS) of composites (**a**) TS contour curve; (**b**) TS contour surface.

The response surface model of panel moisture content and press temperature’s effects on the tensile strength (TS) of composites is illustrated in Figure 8. The figure demonstrates that TS (*Y*_3_) initially increases and subsequently decreases with increasing panel moisture content (*X*_1_) when press temperature (*X*_2_) and press time (*X*_3_) are constant for 10 min. Conversely, TS (*Y*_3_) increases initially and then decreases with increasing press temperature (*X*_2_) when panel moisture content (*X*_1_) is constrained within a narrow range of the experimental level. The parameters of the hot-pressing process were optimised by single-factor experiments and response surface analysis. The results showed that the optimal conditions for achieving the maximum theoretical value of 15 MPa for TS (*Y*_3_) were a panel moisture content of 14%, a press temperature of 170 °C, and a press time of 15 min.

#### 3.3.4. Influence of Moisture Content and Hot-Pressing Parameters on the 2 h Thickness Swelling Rate (2h-TSR) of Composites

The polynomial quadratic equation (Model 4) was solved by employing software SAS v9.0 in conjunction with the response surface methodology. The polynomial quadratic equation of the 2 h thickness swelling rate (2h-TSR) can be obtained as follows (Formula (4)):

(4) $$\begin{array}{cc} Y_{4}= & 1016.91-5.64X_{1}-10.67X_{2}-3.11X_{3}+0.07{X_{1}}^{2}+0.02X_{1}X_{2}\\ & +0.01X_{1}X_{3}+0.03{X_{2}}^{2}+0.01X_{2}X_{3}+0.08{X_{3}}^{2} \end{array}$$

**Figure 9:**
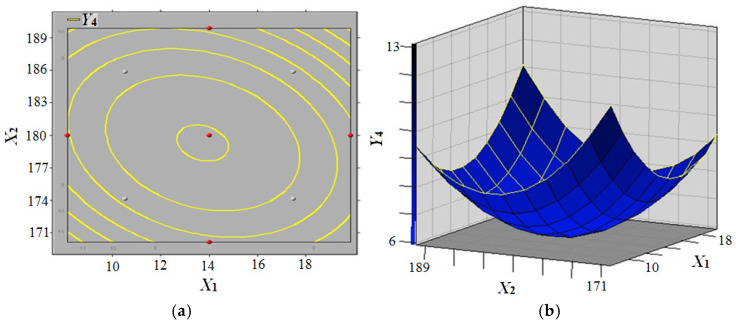
Response surface model of panel moisture content and press temperature effect on the 2 h thickness swelling rate (2h-TSR) of composites (**a**) 2h-TSR contour curve; (**b**) 2h-TSR contour surface.

The response surface model of panel moisture content and press temperature’s on the 2 h thickness swelling rate (2h-TSR) of composites is illustrated in Figure 9. The figure demonstrates a decrease in 2h-TSR (*Y*_4_) initially, followed by an increase, with an increase in panel moisture content (*X*_1_) when press temperature (*X*_2_) and press time (*X*_3_) were constant for 10 min. Conversely, an increase in press temperature (*X*_2_) resulted in a decrease in 2h-TSR (*Y*_4_), when panel moisture content (*X*_1_) was constant within the experimental level. The parameters of the hot-pressing process were optimised through the implementation of single-factor experiments, a design of experiments (DoE) approach, and response surface analysis. The findings indicated that the optimal conditions for achieving the maximum theoretical value of 11% for 2h-TSR (*Y*_4_) were as follows: panel moisture content (*X*_1_) set at 16%, press temperature (*X*_2_) at 170 °C, and press time (*X*_3_) set at 5 min. It was observed that these conditions had to be met for the process to yield the aforementioned result.

## 4. Conclusions

The influence of bamboo powder particle size on the physical and mechanical performance of bamboo/zein biocomposites was a key focus of this study. It was observed that at a particle size of 60 mesh, both the modulus of rupture (MOR) and the modulus of elasticity (MOE) reached their maximum values of 28.72 MPa and 2669.75 MPa, respectively. Furthermore, tensile strength (TS) demonstrated its maximum values of 17.5 MPa at a particle size of 40 mesh, while at a size of 80 mesh, the 2 h thickness swelling rate (2h-TSR) exhibited its minimum value of 5.8%.

The modulus of rupture (MOR), modulus of elasticity (MOE), and tensile strength (TS) achieved their theoretical highest values of 31.86 MPa, 3139.36 MPa, and 15.01 MPa, respectively. These values were achieved under conditions involving panel moisture content ranging from 14% to 16%, press temperature ranging from 170 °C to 175 °C, and press time ranging from 12 min to 15 min. It was observed that the thickness swelling rate (2h-TSR) reached its theoretical lowest value of 11% only when the hot-pressing conditions remained constant and the press time was reduced to 5 min.

## Figures and Tables

**Table 1:** Indicators and parameters of response surface model.

Indicators	Parameters	Properties
Influencing factors	*X*_1_	Moisture content (%)
	*X*_2_	Press temperature (°C)
	*X*_3_	Press time (min)
Response value	*Y*_1_	Modulus of rupture (MOR) (MPa)
	*Y*_2_	Modulus of elasticity (MOE) (MPa)
	*Y*_3_	Tensile strength (TS) (MPa)
	*Y*_4_	2 h thickness swelling rate (2h-TSR) (%)

## Data Availability

Dataset available on request from the authors.
